# BEL β-Trefoil Reduces the Migration Ability of RUNX2 Expressing Melanoma Cells in Xenotransplanted Zebrafish

**DOI:** 10.3390/molecules25061270

**Published:** 2020-03-11

**Authors:** Maria Teresa Valenti, Giulia Marchetto, Massimiliano Perduca, Natascia Tiso, Monica Mottes, Luca Dalle Carbonare

**Affiliations:** 1 Department of Medicine, University of Verona, University of Verona, Ple Scuro, 10, 37100 Verona, Italy; giulia.marchetto@univr.it (G.M.); luca.dallecarbonare@univr.it (L.D.C.); 2 Department of Biotechnology, University of Verona, Strada Le Grazie 15, 37134 Verona, Italy; massimiliano.perduca@univr.it; 3 Department of Biology, University of Padova, I-35131 Padova, Italy; natascia.tiso@unipd.it; 4 Department of Neurosciences, Biomedicine and Movement Sciences, University of Verona, Strada Le Grazie, 10, 37100 Verona, Italy; monica.mottes@univr.it

**Keywords:** BEL β-trefoil, RUNX2, melanoma, zebrafish, lectin

## Abstract

RUNX2, a master osteogenic transcript ion factor, is overexpressed in several cancer cells; in melanoma it promotes cells migration and invasion as well as neoangiogenesis. The annual mortality rates related to metastatic melanoma are high and novel agents are needed to improve melanoma patients’ survival. It has been shown that lectins specifically target malignant cells since they present the Thomsen–Friedenreich antigen. This disaccharide is hidden in normal cells, while it allows selective lectins binding in transformed cells. Recently, an edible lectin named BEL β-trefoil has been obtained from the wild mushroom *Boletus edulis*. Our previous study showed BEL β-trefoil effects on transcription factor RUNX2 downregulation as well as on the migration ability in melanoma cells treated in vitro. Therefore, to better understand the role of this lectin, we investigated the BEL β-trefoil effects in a zebrafish in vivo model, transplanted with human melanoma cells expressing RUNX2. Our data showed that BEL β-trefoil is able to spread in the tissues and to reduce the formation of metastases in melanoma xenotransplanted zebrafish. In conclusion, BEL β-trefoil can be considered an effective biomolecule to counteract melanoma disease.

## 1. Introduction

The incidence of malignant melanoma, a dangerous type of cancer, has been increasing in recent years [1]. It is a highly metastatic tumour generally resistant to apoptosis as well as to chemotherapeutic treatments and local tumour surgery, the standard treatment used. The prognosis for metastatic melanoma is poor, thus there is a need to identify effective molecules to counteract this malignancy. Recent data have suggested that several types of melanoma arise from modifications in genes controlling metabolism, which promote cell proliferation and tumour growth. Genomic sequencing of melanoma cells revealed that this cancer is very heterogeneous: point mutations, deletions and translocations characterize individual tumours [1]. Recently, it has been demonstrated that RUNX2, an important osteogenic transcription factor which is overexpressed in thyroid cancer [2], promotes melanoma cells’ migration and invasion [3,4]. We also found that in melanoma cancer cell lines, this transcription factor is upregulated [5].

New strategies and novel agents are needed to implement current therapies, since diagnoses of metastatic melanoma are frequent and many patients die annually.

It has been demonstrated that lectins can act specifically on malignant cells. This ability is due to the presence of the Thomsen–Friedenreich antigen or T-antigen, a Galβ1-3GalNA disaccharide, in malignant cells. This disaccharide, that is hidden in normal cells [6], allows selective binding of lectins through serines or threonines.

Mushroom-derived lectins have been considered for this purpose, taking advantage of their ligand-binding specificity [6]. For instance, *Agaricus bisporus* lectin, a sugar-binding protein present in edible mushrooms, has been shown to selectively inhibit the proliferation of human malignant epithelial cell lines without toxicity for normal cells [7]. Lectins can be involved in both normal and pathological processes; in vitro studies have suggested to test their suitability for cancer treatment. The rationale to use lectins for cancer therapy arises from their ability to target multiple cellular components, so as to allow cancer treatment by different approaches. It has been demonstrated that lectins are able to affect apoptosis by interacting with signalling pathways involving Bcl-2 and caspase families, p53, PI3K/Akt, ERK, BNIp3, Ras-Raf and ATG families [8]. It is known that the transcription factor RUNX2 is involved in p53 regulation, in the crosstalk between Mek/Erk and PI3K/Akt pathways, and it is also linked to Ras in mechanical stress induction. In addition, RUNX2 induces PI3K/Akt activation in breast cancer cells; RUNX2’s contribution to breast cancer invasiveness via mTORC2 has also been shown. Recently, we found that RUNX2 is overexpressed in melanoma cells and its expression is involved in proliferation and migration processes [9]. In addition, by using the CRISPR/Cas9 system in the A375 melanoma cell line and by performing the xenotransplantation of KO RUNT melanoma cells in zebrafish, we also found that the RUNX2 Runt domain is involved in melanoma proliferation and migration [10]. Then, we also demonstrated that the RUNT domain increases melanoma cells’ angiogenic properties. In particular, a proteomic analysis allowed us to point out RUNT domain involvement in the neoangiogenesis process. Among the deregulated proteins involved in angiogenesis we found fatty acid synthase, chloride intracellular channel protein-4, heat shock protein beta-1, Rho guanine nucleotide exchange factor 1, D-3-phosphoglycerate dehydrogenase, myosin-1c and caveolin-1 [11]. Recently, we purified a lectin with anti-neoplastic properties from the wild mushroom *Boletus edulis* [12]. This protein, named Boletus edulis lectin (BEL) β-trefoil, might represent an interesting therapeutic tool against cancer cells. In addition, our previous study showed simultaneous effects of (BEL) β-trefoil in reducing proliferation as well as migration ability in in vitro-treated melanoma cells. In the present study, we have investigated the effects of BEL β-trefoil against RUNX2- expressing melanoma cells in vivo by using a xenotransplant model of zebrafish.

## 2. Results

### 2.1. BEL β-Trefoil Production

The coding sequence insertion in plasmid pET22b allowed the expression of a recombinant protein with a C-terminal six histidine tag. Using a vector pWaldo-GFP a recombinant protein, called BEL green, fused with Green Fluorescent Protein (GFP) and tagged with eight C-terminal histidines was obtained. Both His-tags were subsequently removed during the purification process before preparing the protein for final use.

**Figure 1:**
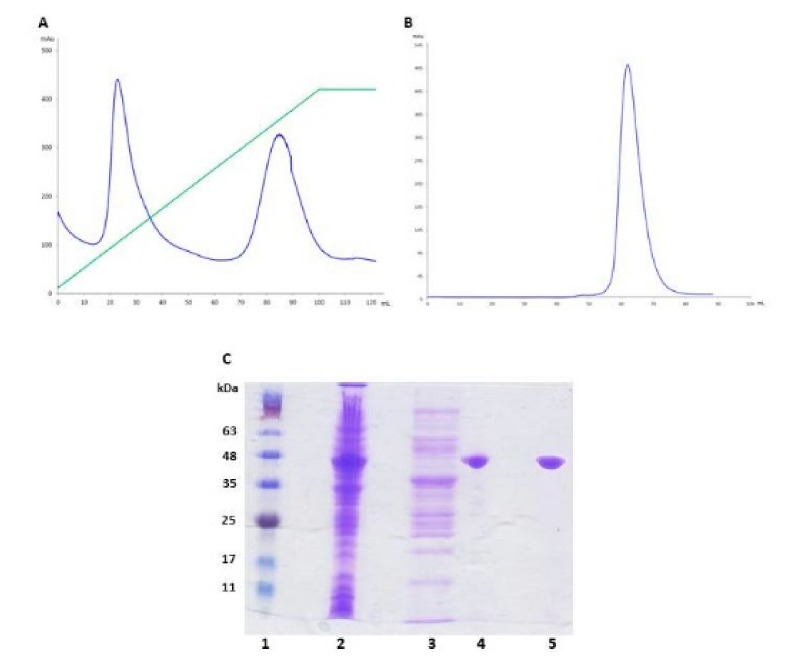
Fluorescent BEL β-trefoil purification. (**A**) Elution profile of fluorescent BEL β-trefoil on the IMAC column. The blue line refers to the protein elution followed spectrophotometrically at 280 nm; the green line represents the increase in the imidazole concentration along the gradient. (**B**) Elution profile of fluorescent BEL β-trefoil on the G75 gel filtration column, recorded at 280 nm. (**C**) SDS-PAGE (12%). Lane 1: molecular mass markers (BlueRay Prestained Protein Marker, Jena Bioscience); lane 2: crude extract from bacteria; lane 3: first peak from the IMAC column, corresponding to nonspecific proteins; lane 4: second peak from the IMAC column, corresponding to the fluorescent BEL β-trefoil; lane 5: pure fluorescent BEL β-trefoil present in the main peak of the G75 chromatography.

The recombinant BEL β-trefoil from pET22b vector was expressed in the BL21 (DE3) *E. coli* strain at 20 °C overnight in liquid Luria Bertani (LB) media; fluorescent BEL β-trefoil was expressed in BL21 C41 (DE3) strain at 20 °C overnight using the Terrific Broth medium to obtain a yield of purification comparable to the non-fluorescent one. Both proteins were purified using an IMAC column, i.e., exploiting the affinity of the his-tag for the nickel ions. They were eluted from the column with a gradient from 10 to 350 mM imidazole and two peaks were obtained for both recombinant proteins as shown in Figure 1, referring to the Fluorescent BEL β-trefoil: the first peak at low imidazole concentration corresponds to unwanted nonspecific contaminants, whereas the second peak contains the rather pure BEL β-trefoil. The next purification step, after the His-tag cleavage, was achieved by a Superdex G75 column, removing the remaining contaminants and obtaining a single band on an SDS-PAGE.

The final yield for the recombinant BEL β-trefoil was 100 mg/L starting from one liter of bacterial culture, whereas that for BEL green was 80 mg/L.

### 2.2. BEL β-Trefoil Injection

**Figure 2:**
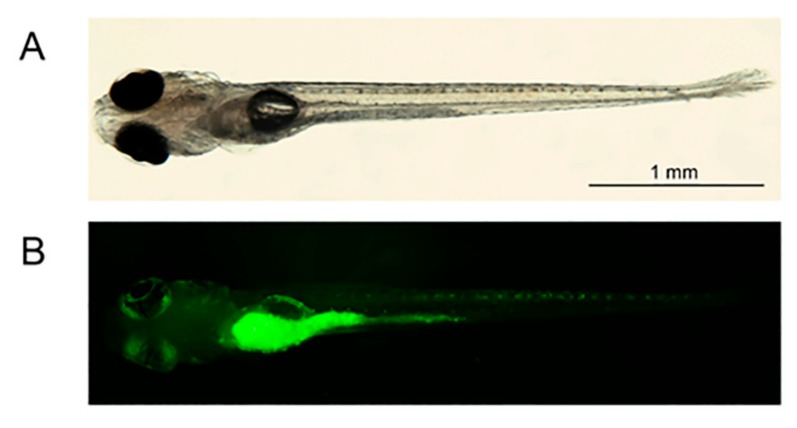
BEL β-trefoil injected in the yolk of zebrafish (N:15) was able to diffuse in tissues. After 5 days of injection (dpi) BEL β-trefoil was still present in zebrafish. (**A**) Phase-contrast photograph of zebrafish (A). (**B**) Fluorescence image showing the GFP-BEL β-trefoil in zebrafish. Magnification 20X.

In order to evaluate the role of BEL β-trefoil in affecting the spreading ability of RUNX2 melanoma cells, we used the in vivo model zebrafish. Zebrafish is considered a useful model to monitor melanoma progression [13]. Melanoma cells were injected with BEL β-trefoil in embryos’ yolk after two days post-fertilization (dpf). As shown in Figure 2, GFP fused BEL β-trefoil (BEL β-trefoil) injected in embryos’ yolk was able to diffuse in zebrafish tissues. Importantly, the number of zebrafish (treated and untreated) at the end of the observation period was reduced exclusively because of mortality (around 60% for all groups).

### 2.3. BEL β-Trefoil Reduces Cells Spreading and Metastases Formation in Vivo

**Figure 3:**
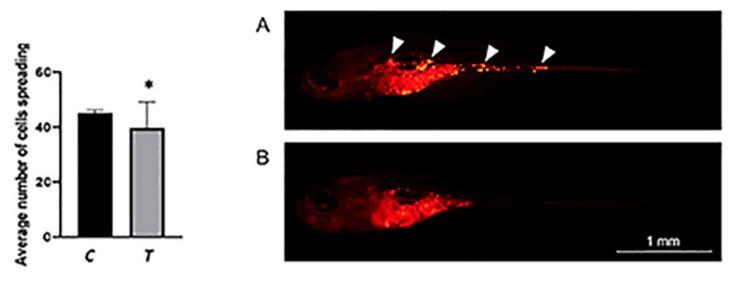
The number of cells spreading was lower in BEL β-trefoil-treated zebrafish (*T*: n45) compared to controls (*C*: n38) (*p* < 0.05). Representative images show untreated (**A**) and BEL β-trefoil-treated (**B**) zebrafish. At 7 dpi, initial metastases (indicated by white arrowheads) are detectable in untreated zebrafish, while most cells are still in the yolk area of treated zebrafish. *P < 0.05; Magnification 20X.

Cell spreading and metastases formation was monitored between the 5th (7 dpf) and 7th (9 dpf) day after the injection of RUNX2-expressing melanoma cells with or without BEL β-trefoil. At 7 days post-injection (7 dpi), the number of cells spreading was lower in BEL β-trefoil-treated zebrafish compared to controls (Figure 3). Importantly, in BEL β-trefoil-treated zebrafish, most of the cells were still in the yolk area (Figure 3). On the contrary, in untreated zebrafish many cells migrated away from the injection area and they formed initial metastases (Figure 3). In addition, the injection of GFP alone in xenotransplanted zebrafish did not affect melanoma cells (Supplementary Figure S1).

**Figure 4:**
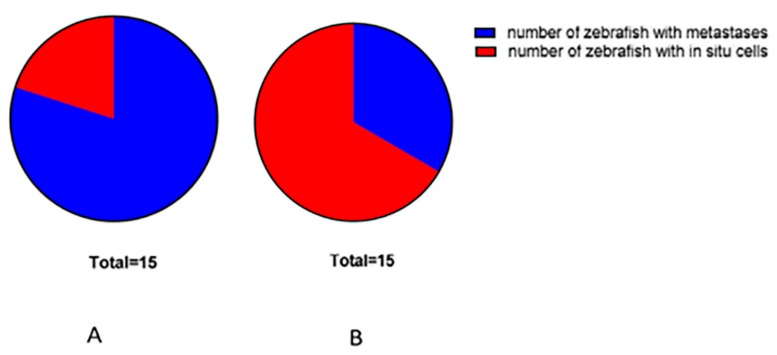
In untreated zebrafish (**A**) melanoma cells migrated in 80% and remained in situ in 20% of individuals, while in the presence of BEL β-trefoil (**B**) melanoma cells migrated in 33% and were still in situ in 67% of transplanted zebrafish.

At 7 dpi (9 dpf), metastases and in situ cells were present in 80% and 20% of untreated zebrafish, respectively. In BEL β-trefoil-treated zebrafish we observed metastases and in situ cells in 33% and 67% of individuals, respectively (Figure 4). These data confirmed the reduced migratory ability of melanoma cells in the presence of BEL β-trefoil.

**Figure 5:**
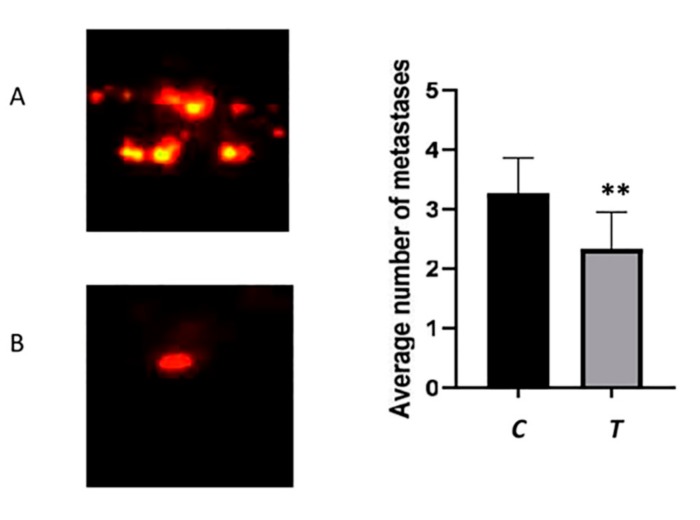
The average number of metastases was higher in untreated zebrafish (**A**) compared to BEL β-trefoil-treated zebrafish (**B**). **p < 0.01. Magnification 200X.

In addition, the average number of metastases was 3.3 for untreated zebrafish, while the number of metastases was 2.3 in BEL β-trefoil-treated zebrafish (Figure 5).

### 2.4. BEL β-Trefoil Reduces Runx2 Expression in Zebrafish

**Figure 6:**
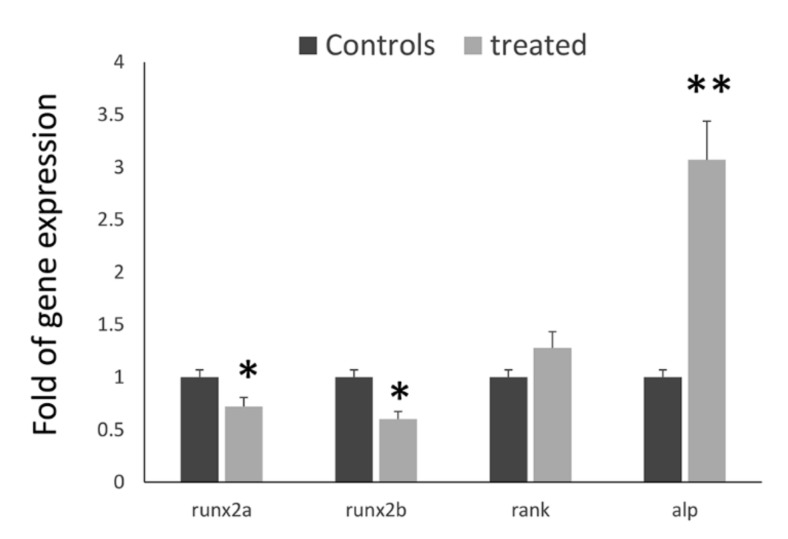
Treatment withBEL β-trefoil reduced the expression levels of both zebrafish endogenous *runx2a* and *runx2b* genes. *p < 0.05. On the contrary, alp expression was upregulated without reaching statistical significance *P < 0.05; **P < 0.01

In order to analyze a possible effect of BEL β-trefoil on the expression of endogenous *runx2*, we treated zebrafish with or without 0.25 µM lectin. At the experimental endpoint (9 dpf), total RNA was collected and the expression of both *runx2a* and *runxb* genes was analyzed. Our analyses showed that lectin treatment affected the expression of endogenous *runx2*, and both *runx2a* and *runx2b* genes were downregulated (Figure 6). Consequently, we analyzed additional genes involved in bone remodeling. In particular, we analyzed the expression of receptor activator of necrosis factor κ-β (rank) gene and of alkaline phosphatase (alp) gene. Our results showed a statistically significant upregulation of alp in lectin-treated zebrafish. We also observed an increased, although not statistically significant, expression of rank.

## 3. Discussion

Recent data suggest that several melanoma types arise from the impairment of gene expression causing cellular proliferation and tumor growth. In general, transcription factors may affect cellular processes and trigger cellular transformation. *RUNX2*, the master transcription factor for osteogenic differentiation, is overexpressed in several tumor tissues, including pancreatic cancer, breast cancer, ovarian epithelial cancer, prostate cancer, lung cancer, thyroid cancer and in osteosarcoma [14]. Lectins are biomolecules holding at least one non-catalytic domain able to bind specific monosaccharides or oligosaccharides [15]. In fact, lectins recognize some sugar moieties, altering cellular physiology and inducing cellular disorders [15]. Therefore, this ability has been exploited to identify altered glycans as well as to induce apoptosis in cancer cells [16]. However, in order to consider these biomolecules as potential tools against cancer, in-depth studies need to be performed. Recently, we demonstrated that BEL β-trefoil affected the viability of melanoma cell lines [9]. Interestingly, we also observed that BEL β-trefoil treatment reduced RUNX2 expression in melanoma cell lines in a dose-dependent fashion [9].

With the aim to explore BEL β-trefoil effects in vivo, we have employed zebrafish xenotransplanted with melanoma cells. Our results have demonstrated that the BEL β-trefoil can spread in the tissues and that it remains detectable for a few days. Importantly, BEL β-trefoil treatment was not toxic for zebrafish, as we observed the same mortality rate in both treated and untreated individuals. In zebrafish transplanted with melanoma cells without BEL β-trefoil treatment, the number of spreading cells as well as the number of metastases was higher compared to treated individuals. In particular, in the presence of the BEL β-trefoil, most of the melanoma cells remained in the injection area (yolk area) and a lower cell number was able to migrate and form metastases, compared to untreated controls. However, BEL β-trefoil affected also the expression of endogenous *runx2*. Runx2 has an important role in bone formation and in bone remodeling [17,18]. In addition, we also observed the upregulation of *alp* and a slight increase in *rank* expression. RANK is a membrane receptor expressed by osteoclasts, bone resorbing cells; it is an important player in the bone remodeling process [19,20]. However, we also observed increased upregulation of *alp*, the gene coding for a glycoprotein involved in osteoblast differentiation [21]. In particular, considering the young age of the zebrafish used, the observed downregulation of *runx2* and upregulation of *alp* may provide evidence of osteogenic maturation rather than a commitment towards osteogenic differentiation. In fact, the expression of *RUNX2* needs to be downregulated in order to allow osteoblast maturation. Recently, we demonstrated that overexpression of RUNX2 impairs bone quality in acromegalic patients [22]. All these findings suggest that the BEL β-trefoil can modulate bone remodeling, even if further studies should be performed to better understand this mechanism. Hence, although the BEL β-trefoil may be considered a beneficial molecule for reducing the metastatic ability of melanoma cells, we suggest that particular attention should be paid to the concurrence of bone disorders. In conclusion, our results confirm the role of the BEL β-trefoil in reducing the migration ability of melanoma cells; to our knowledge, this is the first demonstration of such property in an in vivo model.

## 4. Materials and Methods

### 4.1. Recombinant BEL β-Trefoil Expression and Purification

Recombinant BEL β-trefoil was obtained as previously described [9]. Briefly, the protein coding sequence [12] was inserted into the pET22b plasmid used to transform the *E. coli* BL21 C41 expression strain with a standard heat shock protocol. The protein expression was induced in liquid LB media by adding 0.25 mM IPTG and shacking overnight at 20 °C. Harvested cells were lysed by sonication and the soluble fraction was loaded onto a Nickel–Sepharose column previously equilibrated with buffer 20 mM Tris-HCl pH 7.5, 500 mM NaCl and 10 mM imidazole and the protein was eluted with a gradient of imidazole, from 10 to 350 mM. The histidine tag was cleaved, adding thrombin to the protein solution, and the tag-free protein was concentrated and loaded onto a Superdex G75 column equilibrated with PBS (0.137 M NaCl, 2.7 mM KCl, 10 mM Na_2_HPO_4_, 1.8 mM KH_2_PO_4_, pH 7.4). The protein solution was then sterilized on a 0.2 μm syringe filter.

The fluorescent BEL β-trefoil was obtained by cloning the protein-coding sequence [3] into the pWaldo-GFP plasmid as previously reported [2] in order to obtain the expression of the recombinant BEL β-trefoil fused with a C-terminal GFP. The engineered plasmid was used to transform *E. coli* BL21 DE3 strain and the protein expression was induced in Terrific Broth media by adding 0.25 mM IPTG and shacking overnight at 20 °C.

The chimeric protein was purified using the above protocol of the recombinant BEL β-trefoil alone.

### 4.2. Cell Culture

A375 melanoma cells (purchased from American Type Culture Collection—Rockville, MD, USA) were cultured at 37 °C, 5% CO_2_ and passaged in growth medium DMEM (Invitrogen, Carlsbad, CA), supplemented with 10% FBS (fetal bovine serum) and antibiotics (1% penicillin and streptomycin) and 1% glutamine.

### 4.3. Cell Staining

Cells were detached and harvested by scraping, washed and counted using CountessII (ThermoScientific, Waltham, MA, USA) suspended at a density of 1 × 10^6^ /mL and stained with a lypophilic fluorescent dye that in zebrafish is stable for two weeks according to the literature [23]. Staining with DiI stain (red) Vybrant Cell-Labeling Solution (Molecular Probes, Eugene, Oregon, USA) was performed for 20 min at 37 °C according to the protocol provided by the manufacturer.

### 4.4. Zebrafish Handling and Xenotransplantation

Zebrafish experiments were performed at the Cirsal of the University of Verona, Italy, under ethical authorization 328/2017-PR.

Zebrafish embryos were obtained from natural spawning of *nacre* adults (ZFIN database ID: ZDB-ALT-990423-22), raised according to standard protocols [24] and staged according to Kimmel [25].

For xenotransplantation, embryos were mechanically dechorionated at 2 dpf (days post-fertilization), anesthetized with tricaine 0.16 mg/mL and placed along plastic lanes on a transplantation mould, immersed in 2% methylcellulose/fishwater (all reagents from Sigma-Aldrich, St. Louis, MO, USA).

Stained cells (about 50 cells/embryo) and BEL β-trefoil:GFP (0.25 µM) were loaded in a borosilicate glass capillary needle and microinjected into the yolk, using Eppendorf FemtoJet 4x microinjector and Eppendorf InjectMan micromanipulator (Eppendorf, Hamburg, Germany). Xenotransplanted embryos were grown at 33 °C and monitored daily and documented from 1 day post injection (dpi) up to 1 week (experimental endpoint, 9 dpf). Imaging was performed using Leica M205FA fluorescence microscope (Leica Microsystems, Wetzlar, Germany). Melanoma cells *were* stained with Vybrant DiI (red) stain. We considered the number of stained cells as we reported previously [10]. In particular, stained cells were quantified by Manual Cell Counting using Image J software. To evaluate cellular spreading, cells dispersed throughout the fish body have been considered metastatic cells, whereas cells confined inside the yolk sac area have been considered non-metastatic cells, as previously reported [26]. In particular, we considered those cells that left the perivitellin area as metastatic, whereas we defined those cells which remained in the injection area as “in situ”. Every site with melanoma cells outside the perivitellin area has been considered as a metastasis. We counted these sites manually by using ImageJ. In addition, metastatic individuals were referred to as individuals with metastatic cells, whereas non-metastatic individuals are referred to as individuals with cells observed only in the injection area.

### 4.5. Real Time RT-PCR

At the experimental endpoint (9 dpf), larvae were euthanized and collected. Total RNA was extracted using Qiagen RNeasy kit (Qiagen, Hilden, Germany) following manufacturer’s instructions. RNA concentration was determined using Qubit fluorometer (Invitrogen, Carlsbad, CA, USA).

Reverse transcription with random primers was performed with 1ug of RNA using High-Capacity cDNA Reverse Transcription Kit (Applied Biosystems, Foster City, CA, USA) following manufacturer’s instruction.

Real-Time PCRs were performed in a total volume of 20 μL containing PCR Master with ROX premixed with SYBR Green and 20 ng of cDNA from each sample. The following custom primer sets (Invitrogen, Carlsbad, CA, USA) were used: *runx2a* (fw GACGGTGGTGACGGTAATGG, rv TGCGGTGGGTTCGTGAATA), *runx2b* (fw CGGCTCCTACCAGTTCTCCA, rv CCATCTCCCTCCACTCCTCC), *alp* (fw CAAGAACTCAACAAGAAC, rv TGAGCATTGGTGTTATAC), *rank* (fw GCACGGTTATTGTTGTTA, rv TATTCAGAGGTGGTGTTAT) and as housekeeping gene actb1 (fw CCCAAAGCCAACAGAGAGAA, rv ACCAGAAGCGTACAGAGAGA). For each amount of RNA, three independent experiments with three replicates for each sample were performed. The fold of expression was calculated by the ΔΔCt method by using the TaqMan SDS analysis software as previously reported [27].

### 4.6. Statistical Analysis

Results were expressed as mean ± S.E. Student’s paired *t*-test was used to compare the variation in a variable between two groups. Differences were considered statistically significant when p < 0.05. Statistical analyses were performed using GraphPad Prism software program (version 8.3; GraphPad Software).

## Supplementary Materials

The following are available online. Figure S1: Results observed in controls and GFP treated zebrafish.
