# Supplementary material for: BEL β-Trefoil Reduces the Migration Ability of RUNX2 Expressing Melanoma Cells in Xenotransplanted Zebrafish
Source: Molecules. 2020 Mar 11;25(6):1270. doi: 10.3390/molecules25061270 (PMC7143993; doi:10.3390/molecules25061270)
Supplement: Supplementary file 1 [file molecules-25-01270-s001.pdf]

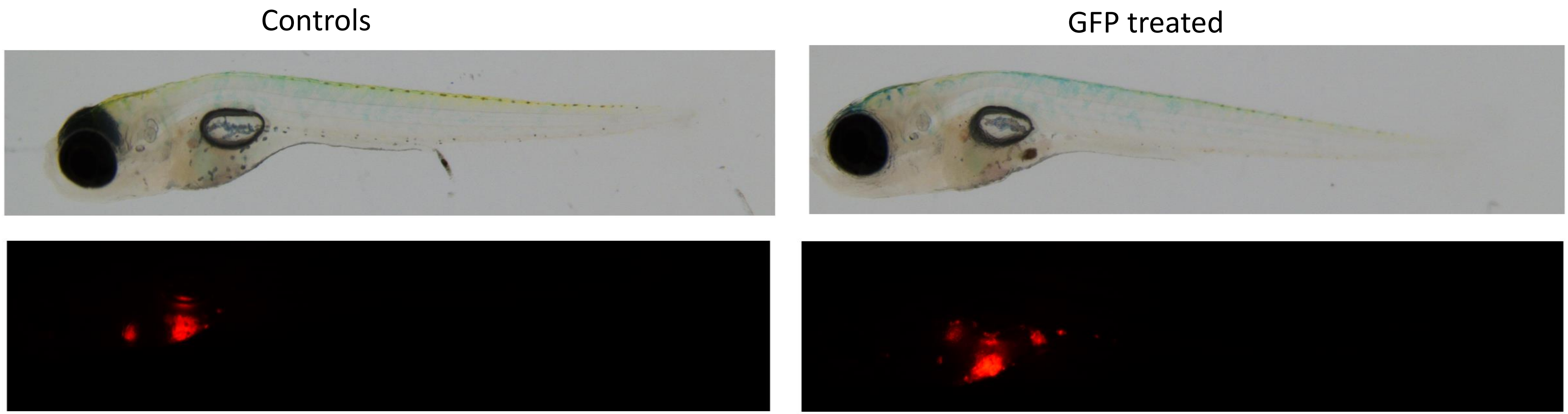

Results in controls and GFP treated zebrafish at 7 dpi

	Controls (N° 6)	GFP injected (N° 6)	P
**Average number of cell spreading**	34 (±10)	38 (±8)	NS
**Number of zebrafish with metastases**	75% (±5%)	80 (±7%)	NS
**Average number of metastases**	2.4 (±0.7)	3 (±1)	NS
